# Diagnosis of Alzheimer's Disease Based on Disease-Specific Autoantibody Profiles in Human Sera

**DOI:** 10.1371/journal.pone.0023112

**Published:** 2011-08-03

**Authors:** Eric Nagele, Min Han, Cassandra DeMarshall, Benjamin Belinka, Robert Nagele

**Affiliations:** 1 Durin Technologies, Inc., New Brunswick, New Jersey, United States of America; 2 Graduate School of Biomedical Sciences, School of Osteopathic Medicine, University of Medicine and Dentistry of New Jersey, Stratford, New Jersey, United States of America; 3 New Jersey Institute for Successful Aging, School of Osteopathic Medicine, University of Medicine and Dentistry of New Jersey, Stratford, New Jersey, United States of America; National Institutes of Health, United States of America

## Abstract

After decades of Alzheimer's disease (AD) research, the development of a definitive diagnostic test for this disease has remained elusive. The discovery of blood-borne biomarkers yielding an accurate and relatively non-invasive test has been a primary goal. Using human protein microarrays to characterize the differential expression of serum autoantibodies in AD and non-demented control (NDC) groups, we identified potential diagnostic biomarkers for AD. The differential significance of each biomarker was evaluated, resulting in the selection of only 10 autoantibody biomarkers that can effectively differentiate AD sera from NDC sera with a sensitivity of 96.0% and specificity of 92.5%. AD sera were also distinguishable from sera obtained from patients with Parkinson's disease and breast cancer with accuracies of 86% and 92%, respectively. Results demonstrate that serum autoantibodies can be used effectively as highly-specific and accurate biomarkers to diagnose AD throughout the course of the disease.

## Introduction

Alzheimer's disease (AD) is an increasingly prevalent and devastating neurodegenerative disease with tremendous social and economic costs, not only to the sufferers but also to caregivers and families. It is the most common cause of dementia worldwide, affecting over 5.4 million people in the United States alone, and has seen a rapidly growing incidence in the aging population [1]. Hallmarks of AD pathology include amyloid-β deposition in neurons, amyloid plaques, tau hyperphosphorylation, neurofibrillary tangles, synaptic loss, and progressive neurodegeneration [2]–[4]. The disease can span decades and is thought to progress unnoticed for 5–10 years before clear symptoms emerge and clinical detection is possible using conventional means [5], [6].

Accurate diagnosis of AD has proven to be difficult to achieve. Current diagnostic practices include neuroimaging techniques, behavioral history assessments, and neuropsychiatric tests [7]. None of these methods by themselves or in combination provide for early detection or yield high accuracy. There has been a great deal of research emphasis on the search for blood-borne biomarkers indicative of AD pathology, but most attempts have found only limited success [7]. Other proposed tests have significant drawbacks in the form of patient discomfort or excessive cost. The Alzheimer's community is still in dire need of a diagnostic method that is accurate, relatively non-invasive, and inexpensive.

Our previous studies have shown that autoantibodies are surprisingly numerous in human sera regardless of age or disease [8], [9]. Suspecting that these autoantibodies may play a role in neurodegenerative diseases, we sought to determine if the presence of ongoing pathology causes changes in the spectrum of autoantibodies present in the serum. If so, then perhaps these changes could be used to identify specific autoantibodies that are useful as diagnostic indicators or biomarkers. Given the large number of autoantibodies present in human sera, we utilized high-throughput protein microarray technology to assess individual autoantibody expression profiles. We searched for disease group- and control group-specific variations in autoantibody expression patterns in an effort to identify potentially useful diagnostic biomarkers. Our results show that autoantibody expression profiles, determined using protein microarray technology, can be used to select a relatively small panel of useful autoantibody biomarkers that can detect the presence of specific diseases such as AD with great accuracy using only a small sample of blood.

## Materials and Methods

### Ethics Statement

Approval for the use of blood samples for this study was obtained from the UMDNJ-Stratford Institutional Review Board.

### Patient Samples

Serum samples from 50 AD subjects and 40 non-demented controls (NDC) were obtained from *Analytical Biological Systems, Inc*. (Wilmington, DE). 30 breast cancer (BC) serum samples and 29 Parkinson's disease (PD) serum samples were obtained from *Asterand, Inc*. (Detroit, MI). To represent different disease stages reflecting disease severity, our AD serum pool contains samples with Mini-Mental State Examination (MMSE) scores ranging from 2–24. All samples were handled by standard procedures and stored at −80°C. Diagnosis of AD was based on a medical evaluation, neuropsychiatric testing, and on the National Institute of Neurological and Communicative Disorders and the Alzheimer's Disease and Related Disorders Association criteria. Demographic characteristics of the study population are shown in Table 1.

**Table 1:** Demographics of serum donors.

Group	n	Age		Sex	MMSE
		Mean	Range	(% male)	
**Alzheimer's disease**	50	78.5	61–97	40%	2–24
**–Earlier-stage** 1	35	78.7	61–97	43%	15–24
**–Later-stage** 2	15	78.0	65–94	33%	2–14
**Non-demented Controls**	40	40.4	19–86	82%	–
**–Older Control**	20	57.7	51–86	100%	–
**–Younger Control**	20	24.7	19–30	65%	–
**Parkinson's disease**	29	74.0	53–88	55%	–
**Breast Cancer**	30	46.7	32–54	0%	–

1 *Earlier-stage: AD patients with MMSE≥15.*

2 *Later-stage: AD patients with MMSE<15.*

### Human Protein Microarrays

To identify autoantibodies in human sera, we used *Invitrogen's* ProtoArray v5.0 Human Protein Microarrays (Cat. *No*. PAH0525020, *Invitrogen*, Carlsbad, CA, USA), each containing 9,486 unique human protein antigens (www.invitrogen.com/protoarray). All proteins have been expressed as GST fusion proteins in insect cells, purified under native conditions, and spotted in duplicate onto nitrocellulose-coated glass slides. All arrays were probed and scanned according to the manufacturer's instructions using commercially prepared reagents. Briefly, microarray slides were blocked (Blocking Buffer, Cat. *No. PA055*, *Invitrogen*) and then incubated with serum samples, diluted 1∶500 in washing buffer. After washing, the arrays were probed with anti-human IgG (H+L) conjugated to AlexaFluor 647 (Cat. *No.* A-21445, Invitrogen). Arrays were then washed, dried, and immediately scanned with a GenePix 4000B Fluorescence Scanner (*Molecular Devices*, Sunnyvale, CA, USA).

### Dot Blot Analysis

One µl volumes of purified recombinant human FRMD8 (0.2 µg/µl) and PTCD2 (0.1 µg/µl) proteins (Cat. No. TP307879 and TP315253, OriGene Technologies, Inc., Rockville, MD, USA), were manually pipetted onto a nitrocellulose membrane. The proteins were blocked in a 5% non-fat milk PBS-Tween solution for one hour at room temperature (RT). The proteins were then probed with human serum samples diluted 1∶2000 for one hour at RT. All sera were identical to those used to probe the human protein microarrays. The dot blots were probed with anti-human IgG (H+L) HRP conjugate antibody (Cat. No. 31410, Thermo Fisher Scientific Inc., Pittsburgh, PA, USA) for one hour at RT, incubated with ECL reagent (Cat. No. 34096, Thermo Fisher Scientific Inc., Pittsburgh, PA, USA) for one minute, and then exposed to X-ray film at various intervals.

### Data Analysis

The fluorescence data for each microarray was acquired by *Genepix Pro* analysis software after scanning, and then synced with Invitrogen's lot-specific *Genepix Array List* (GAL) files. The resulting *Genepix Results* (GPR) files were then imported into Invitrogen's *Prospector 5.2* for analysis. All data is MIAME compliant and have been deposited in NCBI's Gene Expression Omnibus and are accessible through GEO Series accession number GSE 29676. The “group characterization” and “two - group comparison” features in the *IRBP Toolbox* allowed for M-statistical analysis of autoantibody expression. Sorting detectable autoantibodies by difference of prevalence between AD and NDC groups in descending order, we selected the top 10 as our potential diagnostic biomarkers.

The selected biomarkers were re-verified as significant by *Predictive Analysis for Microarrays* (*PAM*) – an independent algorithm relying on nearest shrunken centroid analysis to identify proteins acting as significant class-differentiators. The predictive classification accuracy of the identified biomarkers was tested with *Random Forest* (*RF*) using the default settings, another significance algorithm run as an *R* package (v 2.12.1). In *RF*, partitioning trees are built by successively splitting the samples according to a measure of statistical impurity at a given node until terminal nodes are as homogenous as possible. Classification accuracy for a given set of diagnostic biomarkers is reported in a confusion matrix and misclassification as an Out-Of-Bag (OOB) error score.

## Results

### Protein Microarrays Reveal That Autoantibodies Are Numerous in Human Serum

To detect autoantibodies in sera, we probed protein microarrays with individual serum samples (n = 149) (Table 1). Results using the standard Chebyshev Inequality p-value threshold of 0.05 suggest an average of over one thousand different autoantibodies per serum sample; although the number varied widely from one individual to the next (n = 149, 1115±1096) (Table 2). This, along with our previous work showing the presence of abundant autoantibodies via western analysis [8], [9], provides strong support for a large number of autoantibodies in human sera. It appears that this may be a generally unappreciated feature of the blood, with a function that remains to be elucidated.

**Table 2:** Estimate of autoantibodies per sample group.

Sample Group	(n)	Median	σ	Range
**All Samples**	149	920	1096	0–6389
**Alzheimer's disease**	50	969.25	770	0–3311
**–Earlier-stage**	35	826.5	672	0–2805
**–Later-stage**	15	1321.5	865	110–3311
**Non-demented controls**	40	982	965	0–3585
**–Older Controls**	20	1066.25	896	32–2675
**–Younger Controls**	20	942.5	1050	0–3585
**Parkinson's disease**	29	539.5	762	0–2585
**Breast Cancer**	30	884.5	1723	5–6389

### Selection of Autoantibody Biomarkers for AD Diagnosis

**Figure 1:**
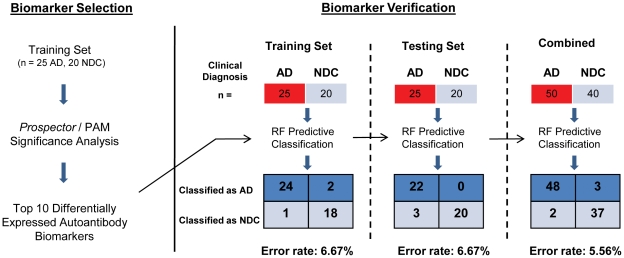
Biomarker selection and Training / Testing Analysis. Before biomarker selection, our total sample pool was split into two randomized groups: the Training Set and Testing Set. *Prospector* and *PAM* statistical analyses were performed on the Training Set to identify the top 10 most significant autoantibody classifiers of AD and NDC. We then verified the diagnostic accuracy of these selected biomarkers by using *Random Forest* to predict sample classification in the Training Set, Testing Set, and then both sets combined.

A total of 90 human serum samples (50 AD and 40 NDC) were randomly assigned to either a Training or Testing Set composed of 25 AD and 20 NDC sera each; both containing equal proportions of earlier- and later-stage AD samples as well as older and younger controls. To identify potential diagnostic autoantibodies, we probed protein microarrays, each containing 9,486 antigens, with Training Set sera and analyzed the data as described in the methods section (Fig. 1). *Prospector* analysis software determined that 451 autoantibodies had a significantly higher prevalence in the AD group than in the NDC group (p<0.01). We selected the 10 biomarkers that demonstrated the largest difference in group prevalence between AD and NDC to serve as our diagnostic indicators (Table 3). As an independent verification of the 10 biomarkers selected, we also utilized *Predictive Analysis for Microarrays* (*PAM*) to re-evaluate our data [10]. *PAM* confirmed that the 10 biomarkers originally selected by *Prospector* were among the most significant classifiers of AD and NDC.

**Table 3:** Identity and significance of 10 ad vs. Ndc diagnostic biomarkers.

Database ID	Description	Prevalence in AD	Prevalence in Control	p
**NM_024754.2**	Pentatricopeptide repeat domain 2 (PTCD2)	94.23%	14.29%	8.03E-14
**BC051695.1**	FERM domain containing 8 (FRMD8)	73.08%	4.76%	4.06E-13
**NM_018956.2**	Chromosome 9 open reading frame 9 (C9orf9)	82.69%	14.29%	3.30E-09
**NM_002305.2**	Lectin, galactoside-binding, soluble, 1 (galectin 1) (LGALS1)	65.39%	9.52%	3.76E-08
**NM_000939.1**	Proopiomelanocortin (adrenocorticotropin/ beta-lipotropin/ alpha-melanocyte stimulating hormone/ beta-melanocyte stimulating hormone/ beta-endorphin) (POMC), transcript variant 2	65.39%	11.91%	1.18E-05
**NM_003668.2**	Mitogen-activated protein kinase-activated protein kinase 5 (MAPKAPK5), transcript variant 1	71.15%	11.91%	8.91E-09
**BC033758.1**	Centaurin, alpha 2 (CENTA2)	82.69%	23.81%	5.27E-08
**NM_014280.1**	DnaJ homolog subfamily C member 8	78.85%	11.91%	9.49E-12
**NM_024668.2**	Ankyrin repeat and KH domain containing 1 (ANKHD1), transcript variant 3	73.08%	14.29%	1.05E-06
**NM_023937.1**	Mitochondrial ribosomal protein L34 (MRPL34), nuclear gene encoding mitochondrial protein	73.08%	16.67%	3.15E-05

### Verification of Biomarkers via Training and Testing Set Analyses

To assess the Training and Testing set classification accuracies of the 10 selected biomarkers, we used *Random Forest* (*RF*) [11]. *RF* is a statistical algorithm which creates voting classes of decision-making trees to evaluate the significance of each marker and classify samples. Using our 10 biomarkers to “diagnose” the Training Set (n = 45; 25 AD and 20 NDC), *RF* had an overall accuracy of greater than 93% [Out-of-Bag (OOB) Error 6.67%, a positive predictive value (PPV) of 92.3%, and a negative predictive value (NPV) of 94.7%]. When the 10 biomarkers were used to classify the Testing Set sera (n = 45; 25 AD and 20 NDC), which played no part in the biomarker selection process, *RF* distinguished AD samples from NDCs with a similar accuracy (prediction error of 6.67%, PPV of 100.0%, and NPV of 87.0%).

### Biomarker Performance in Different Sample Demographics

When the 10 autoantibody biomarkers were used to classify all AD and NDC samples combined (n = 90; 50 AD, 40 NDC) using *RF*, they did so with a 96.0% sensitivity and 92.5% specificity. We also tested their performance in classifying samples from different demographics: earlier-stage AD, later-stage AD, older controls, and younger controls. The 10 biomarkers classified samples with over 90% accuracy in all subgroups tested (Table 4). AD samples were correctly differentiated from younger controls with high and consistent accuracy, a common-sense indication of biomarker credibility.

**Table 4:** Diagnostic accuracies of selected biomarkers.

	AD (n = 50) vs.					Earlier-stage AD (n = 35) vs.		Later-stage AD (n = 15) vs.	
	All NDC	Older Control	Younger Control	PD*	Breast Cancer	All NDC	Older Control	All NDC	Older Control
	n = 40	n = 20	n = 20	n = 29	n = 30	n = 40	n = 20	n = 40	n = 20
**Sensitivity %**	96.0	98.0	98.0	90.0	98.0	97.1	97.1	86.7	93.3
**Specificity %**	92.5	85.0	90.0	79.3	83.0	92.5	90.0	97.5	90
**PPV%**	94.1	94.2	96.1	88.2	90.7	91.9	94.4	92.9	87.5
**NPV %**	94.9	94.4	94.7	82.1	96.2	97.4	94.7	95.1	94.7

*The biomarkers used for this classification are those of *Table 5* *; all others are the biomarkers identified in* *Table 3* *.*

### Distinction of AD From Other Diseases

One must be careful in the creation of a biomarker diagnostic to ensure disease specificity. Therefore, we sought to differentiate AD from other non-neurological and neurological diseases. We acquired 30 breast cancer serum samples and used our 10 selected diagnostic biomarkers to differentiate them from the 50 AD samples. *RF* reported an OOB Error of 7.5% (PPV and NPV of 90.7% and 96.2%, respectively). These results are similar to those of the AD versus NDC trials above and demonstrate no diagnostic bias toward general disease.

We next sought to determine if it is possible to differentiate between two closely related neurodegenerative diseases. For this, we selected Parkinson's disease (PD) because it shares much in common with Alzheimer's pathology [12], [13]. There is also a significant overlap (22%∼48%) at the pathological and clinical levels, making it difficult to clearly distinguish these two diseases by conventional means alone [14], [15]. Again, we utilized *Prospector*, *PAM*, and *RF* to identify the most significant disease classifiers. We determined that by using only five diagnostic biomarkers (Table 5), it was possible to differentiate AD samples from PD samples with over 86% accuracy (sensitivity = 90.0%, specificity = 79.3%). To our knowledge, this is the highest efficiency ever achieved with blood biomarkers to distinguish these closely related neurodegenerative diseases [15], [16].

**Table 5:** Identity and significance of five AD vs. PD diagnostic biomarkers.

Database ID	Description	Prevalence in AD	Prevalence in PD	*p*
**BC051695.1**	FERM domain containing 8 (FRMD8)	9.62%	45.16%	5.93E-04
**NM_003177.3**	Spleen tyrosine kinase (SYK)	19.23%	70.97%	1.35E-05
**BC019015.2**	Mediator complex subunit 29 (MED29)	9.62%	61.29%	1.61E-06
**BC003551.1**	Transglutaminase 2 (C polypeptide, protein-glutamine-gamma-glutamyltransferase) (TGM2)	13.46%	61.29%	9.67E-05
**BC001755.1**	Leiomodin-1	26.92%	70.97%	6.84E-05

### Dot Blot Confirmation of Potential Biomarkers

**Figure 2:**
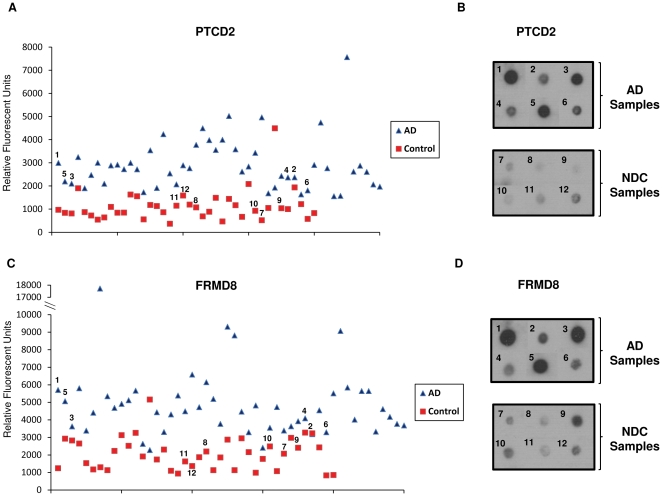
Differential Expression of PTCD2 and FRMD8 autoantibodies in AD and NDC sera. Microarray fluorescence values reflecting individual serum autoantibody titers demonstrate a difference in the expression of anti-PTCD2 and anti-FRMD8 in AD (n = 50) and NDC (n = 40) sera (a,c). This difference was confirmed in independent dot blots that assessed AD and NDC sera reactivity to purified PTCD2 and FRMD8 protein antigens (b,d).

To further validate the differential expression of autoantibodies detected with human protein microarrays, we carried out a comparative dot-blot analysis using commercially-obtained, purified native proteins. We selected two of the most potent differentiating antigens identified, PTCD2 and FRMD8, and sought to verify their reactivity. The two proteins were spotted onto nitrocellulose membrane and probed with identical sera to that used on the microarrays. Results from both AD and NDC sera show strong agreement in the relative intensities of the immunoreaction in protein microarrays and dot blots (Fig. 2). The majority of AD sera reacted intensely to purified PTCD2 and FRMD8 protein, while most control sera showed a weak or no reaction (Figs. 2b, 2d). Dot blot assays independently confirmed that anti-FRMD8 and anti-PTCD2 antibodies were more predominant in AD sera than in NDC sera, and so are potentially useful as diagnostic biomarkers. Continued efforts are needed to independently confirm the remaining biomarkers.

## Discussion

The identification and development of blood-borne biomarkers for accurate diagnosis and early detection of AD has long been a central goal. In the present study, we used human protein microarrays to confirm our earlier discovery using western analysis that autoantibodies are unexpectedly numerous and perhaps universally present in human sera. We also demonstrated that the presence of disease can cause characteristic alterations in serum autoantibody profiles such that specific autoantibodies and their cognate antigens can be used effectively as diagnostic biomarkers of ongoing disease. Lastly, we demonstrate that accurate detection and diagnosis of AD from a blood sample is possible with only a small subset of these autoantibody biomarkers.

### Autoantibodies Are Numerous in Human Serum

The number of autoantibodies detected in sera using protein microarrays was found to be surprisingly high, averaging over one thousand per sample but displaying wide individual variations. Ascertaining the true number of autoantibodies in individual serum samples is difficult for several technical reasons. In addition, any determination of this number employing the protein microarrays used here will, of course, be an underestimate, since the available autoantigens represent only about one third of the estimated human proteome. Regardless of these limitations, it is clear that the number of autoantibodies in a single serum sample is much higher than previously thought. The function of such a large number of autoantibodies is unknown. We suspect that they have some hitherto unrecognized, but important, role that remains to be elucidated.

### An AD diagnostic Based on Detection of Tell-tale Autoantibody Profiles

The present study demonstrates that AD can be linked to characteristic alterations in serum autoantibody expression profiles. These changes allow for the identification and selection of specific autoantibodies that can serve as diagnostic biomarkers. As exemplified above, with only 10 autoantibody diagnostic biomarkers, AD serum samples are readily distinguished from NDC sera with a sensitivity of 96.0% and a specificity of 92.5%. The fact that these serum autoantibody biomarkers show similar patterns of reactivity in dot blots spotted with purified, native proteins further confirms the validity of the immunoreactions on protein microarrays. We also tested the efficacy of our chosen biomarkers in differentiating multiple sample demographics of varying age and MMSE-score. We were able to distinguish AD patients from controls with over 90.0% accuracy in all subgroup comparisons. This successful classification of AD across the full range of available MMSE scores suggests that this approach is useful for AD diagnosis throughout the full course of the disease, and may also be useful for early detection, perhaps including patients with mild cognitive impairment and pre-symptomatic disease.

Future work involving more samples should extend our understanding of autoantibody expression and further optimize diagnostic success. Many of the samples used in this study were from living donors, and so their AD was diagnosed using standard clinical practices [17]. The highest accuracy claimed by these methods is roughly 90% [18]–[22] – thus, there is a possibility of inaccurate sample labeling. As our efforts continue with more samples and *post-mortem* validation of AD, the accuracies reported above should reflect a corresponding increase.

### Multiplicity of the AD Diagnostic Panel

Aside from the discovery of so many autoantibodies being present in the blood, another unexpected finding was that many of these autoantibodies are differentially expressed in the AD and NDC groups, and so are potentially useful as diagnostic biomarkers. In fact, *Prospector* identified 199 differentiating autoantibodies with a p-value of less than 0.0001 and group prevalence differences of over 40%. Importantly, this evaluation of significance was duplicated by the other statistical algorithms used here, *PAM* and *RF*. Most autoantibodies considered significant in one program were repeatedly selected as significant diagnostic biomarkers by the other two programs. This finding suggests that many combinations of autoantibody biomarkers can be used to successfully distinguish AD sera from NDC sera with varying accuracies. Paradoxically, this multiplicity of diagnostic indicators often complicates bioinformatic analyses. The apparent inconsistency of biomarkers selected by algorithms like *RF* has been extensively discussed by others [23], [24]. This has been blamed on many features of biological data, including number of variables and relative “closeness” of values. However, as reported above, we find that there are many relevant autoantibody biomarkers with diagnostic potential that make possible multiple “solutions” to a single diagnostic question. Thus, in this case, we contend that what often appears as inconsistency in this type of analysis might, in fact, simply be the selection of an equally viable set of biomarkers by the significance analysis programs.

### Hypothesis Underlying the Generation of Diagnostic Autoantibodies

The underlying reason for the presence and abundance of autoantibodies in human sera, especially in younger and healthy individuals, is unknown. Although some autoantibodies may be vestiges of past diseases and reflect a history of immunological activity, it is clear that many are also present as a result of ongoing disease. We suggest that active diseases, resulting in cell damage and death, cause the production and release of antigenic cellular products. In the case of AD, the somewhat selective early loss of pyramidal neurons provides a chronic, yet specific, source of such breakdown products. These products enter the cerebrospinal fluid, diffuse into the blood and lymph, with some presumably acting as antigens to elicit an immune response. We propose that this response leads to the production and appearance of a relatively large number of autoantibodies in the blood. Since many diseases exhibit damage to specific cell and tissue types, the biomarker discovery strategy described here could conceivably be applicable to the development of successful diagnostics for a wide variety of diseases.

### Potential Benefits of Antigen Identification

One further advantage of using protein microarrays to detect disease-related autoantibodies in sera is that their antigen targets also become known. This knowledge may prove to have therapeutic implications, especially if it sheds new light on disease-relevant pathways. Such information could be used to develop therapies that combat pathology by targeting important members of these pathways. Currently, little is known about the functions of most of the antigens identified here as targets of the autoantibody biomarkers for AD. Many of them are explicit only at the genetic level as elucidated by efforts in creating comprehensive cDNA libraries [25]. As more is learned about the functions of autoantibodies in the sera and their targets, we anticipate that a better understanding of autoantibody profiles will eventually yield significant therapeutic benefits.

### Conclusion

The development of a reliable and accurate blood test for AD will have profound clinical impact. The identification and use of a small panel of AD autoantibody biomarkers shown here has a diagnostic sensitivity of 96.0% and specificity of 92.5% using available samples. The relative non-invasiveness, low cost, and dynamism of protein microarrays make a diagnostic of this kind well-suited for incorporation into routine health care. We hope that with a diagnostic such as this, accessible early screening methods can be established so that patients will be better positioned to avail themselves of effective therapies as they arise.
